# Knowledge, perception and practices about malaria, climate change, livelihoods and food security among rural communities of central Tanzania

**DOI:** 10.1186/s40249-015-0052-2

**Published:** 2015-04-24

**Authors:** Benjamin K Mayala, Carolyn A Fahey, Dorothy Wei, Maria M Zinga, Veneranda M Bwana, Tabitha Mlacha, Susan F Rumisha, Grades Stanley, Elizabeth H Shayo, Leonard EG Mboera

**Affiliations:** National Institute for Medical Research, Dar es Salaam, Tanzania; Georgetown University, Washington, DC USA; Catholic University of Health and Allied Sciences- Bugando, Mwanza, Tanzania

**Keywords:** Malaria, Agriculture, Climate change, Food insecurity, Knowledge, Practice, Tanzania

## Abstract

**Background:**

Understanding the interactions between malaria and agriculture in Tanzania is of particular significance when considering that they are the major sources of illness and livelihoods. The objective of this study was to determine knowledge, perceptions and practices as regards to malaria, climate change, livelihoods and food insecurity in a rural farming community in central Tanzania.

**Methods:**

Using a cross-sectional design, heads of households were interviewed on their knowledge and perceptions on malaria transmission, symptoms and prevention and knowledge and practices as regards to climate change and food security.

**Results:**

A total of 399 individuals (mean age = 39.8 ± 15.5 years) were interviewed. Most (62.41%) of them had attained primary school education and majority (91.23%) were involved in crop farming activities. Nearly all (94.7%) knew that malaria is acquired through a mosquito bite. Three quarters (73%) reported that most people get sick from malaria during the rainy season. About 50% of the respondents felt that malaria had decreased during the last 10 years. The household coverage of insecticide treated mosquito nets (ITN) was high (95.5%). Ninety-six percent reported to have slept under a mosquito net the previous night. Only one in four understood the official Kiswahili term (*Mabadiliko ya Tabia Nchi*) for climate change. However, there was a general understanding that the rain patterns have changed in the past 10 years. Sixty-two percent believed that the temperature has increased during the same period. Three quarters of the respondents reported that they had no sufficient production from their own farms to guarantee food security in their household for the year. Three quarters (73.0%) reported to having food shortages in the past five years. About half said they most often experienced severe food shortage during the rainy season.

**Conclusion:**

Farming communities in Kilosa District have little knowledge on climate change and its impact on malaria burden. Food insecurity is common and community-based strategies to mitigate this need to be established. The findings call for an integrated control of malaria and food insecurity interventions.

**Electronic supplementary material:**

The online version of this article (doi:10.1186/s40249-015-0052-2) contains supplementary material, which is available to authorized users.

## Multilingual abstracts

Please see Additional file 1 for translations of the abstract into the six official working languages of the United Nations.

## Background

Health, agriculture, and food security are interdependent, whereby failure in one has negative effects on the others. These interactions operate in a vicious cycle: illness and death, such as that caused by malaria, detract from farm worker productivity, which leads to low harvests, thereby resulting in food insecurity [1]. This, in turn results in malnutrition and weakened immune systems that are more susceptible to illness [2,3]. Further links between agriculture, food security, and malaria show that these issues should be addressed in tandem rather than as stand-alone issues. Many developing countries rely on agriculture as a significant portion of the national economy, therefore affecting the government’s ability to finance public services including healthcare [4]. Looking ahead, agriculture will have an increased role in health as climate change takes a toll on agricultural yields and the individuals and economies that affect farmers’ livelihoods. On the other hand, some agricultural practices also pose health hazards, including providing environments for mosquito breeding and thus contributing to malaria [5,6].

Malaria control, agricultural development, food security are all high on global health and development agendas. Reducing the incidences of hunger and malaria are among the specific aims of the Millennium Development Goals. International funding for malaria control rose to a peak of US$2 billion in 2011; while the 2006–2008 world food crisis brought food security to the forefront and has set policymakers to work figuring out how to manage volatile world food prices [4,7,8]. Despite the attention and funding given to agriculture, climate change, food security, and malaria, little research has been done on the links between all four drivers at community level. Studies on the relationship between food security, malaria and climate change are few [9,10]. Links between agriculture and food security are intuitive, yet understanding of this dynamic is still being developed; meanwhile, studies have only recently begun on the interactions between agriculture and malaria [5,11-13]. Though the four drivers are considered separate development issues in need of independent intervention strategies, there are reasons to assume that agriculture, climate change, food security, and malaria overlap and affect each other. Understanding these links is imperative to health and progress particularly in Africa, where 85% of the countries are endemic for malaria and where 90% of global deaths from the disease occur [14].

Tanzania is an important place to explore the links between agriculture, climate change, food security and malaria. Malaria is considered the most important public health problem and the focus of major public health interventions [15]. Chronic malnourishment and food insecurity is also a problem in Tanzania, made even more serious by escalating food prices around the world [7]. Meanwhile, agriculture accounts for 45% of Gross Domestic Product and employs 75% of the population [16]. With the majority of agriculture being rain fed, crops are highly vulnerable to climatic changes [16]. Climate change events including floods, droughts have already had drastic effects on lives and destroyed crops [7] and have been associated with a number of communicable diseases, including malaria [9].

Understanding the interactions between malaria, food security and climate change in Tanzania is of particular significance when considering that they are the major sources of livelihoods and illness. The objective of this study was to determine knowledge, perceptions and practices as regards to malaria, climate change, livelihoods and food insecurity in a rural farming community in central Tanzania.

## Methods

### Study area

The study was carried out in Kilosa District (5°55’ -7°53’ S; 36°30’ - 37°30 E), located in central Tanzania about 300 km west of Dar es Salaam, and about 70 km from Morogoro town. The district has a total surface area of about 14,400 km^2^ and a population of 489,513 people living in 105,635 households with an average household size of 4.6 people.

**Figure 1:**
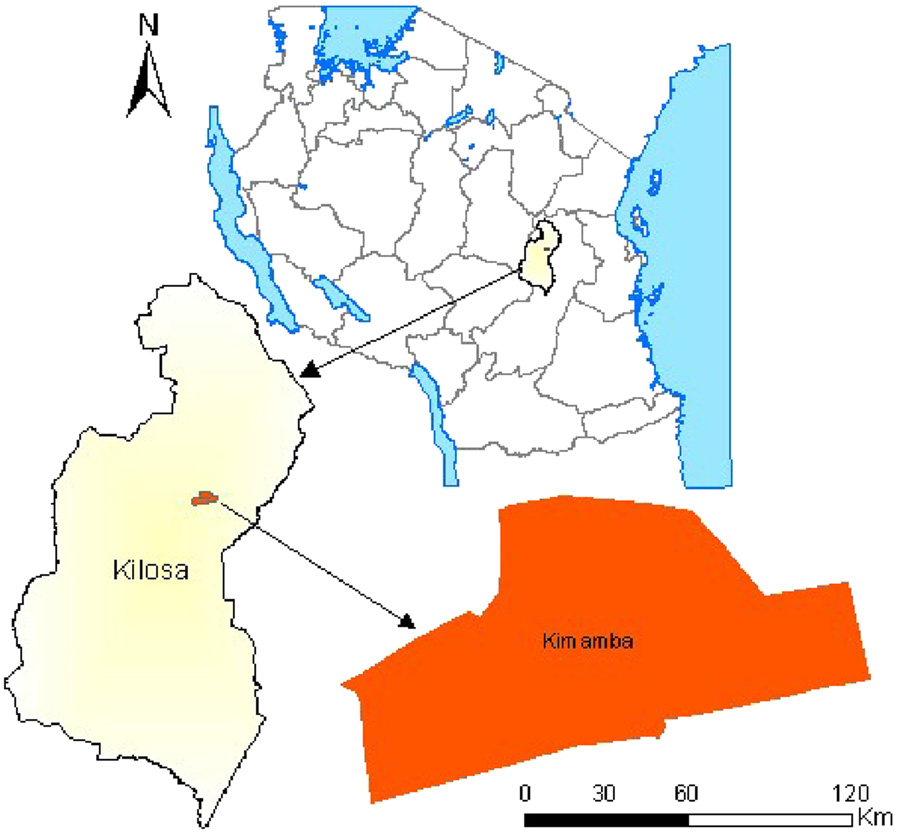
Map of Tanzania showing the study area in Kilosa District.

Kimamba Ward of Kilosa District was purposely selected for this study. Kimamba is located at about 21 km from Kilosa District capital and 59 km from Morogoro Regional capital (Figure 1). The village has a total population of 7,810 whereby 4,102 are women and 3,708 are men. Kimamba is in the lowland and flatland area characterized by few valleys and hills. The vegetation cover consists of bushes, short grasses and scattered trees. The inhabitants cultivate mainly maize and rice. The area is characterized by large sisal farms. A number of inhabitants are employees and labourers in the sisal estates.

The area experienced severe flooding in 2009/2010, which has been attributed to climate change coupled with environmental degradation (http://www.ifrc.org/docs/appeals/10/MDRTZ0101.pd). During the 2009/2010 floods, 1076 acres of maize farming land were flooded, covered in sand and mud, and are no longer fit for future farming (Kilosa District Council, 2010 unpubl.). Resultant crop failures due to flooded fields resulted in a loss of income and food insecurity for farming communities. The impact was huge to some extent that some families were still displaced and were living outside their home areas during the study period in November 2011.

### Study design

This was a household-based study that involved face-to-face interviews with heads of households (≥18 years old). A purposive sampling was used to get a minimum sample size of households to be involved in the study. The sample was calculated using the using the World Health Organization Sample Size Determination in Health Studies [17] assuming a 41% prevalence of malaria, with a 95% confidence interval and a 5% relative precision. The sample size was then adjusted by a refusal rate of 5% which gives a total of 390 households. The household to participate in the study were selected with the criterion that crop production was contributing about 50% of the household economic activities.

### Data collection

The study participants were recruited and interviewed at their homes. Participants were interviewed in Kiswahili using a semi-structured questionnaire. Household information was collected in three broad categories: (i) Malaria knowledge and perception, which included the cause of malaria, the symptoms, the season people get malaria most, treatment and prevention, and whether or not malaria has decreased in the study area during the past 10 years; (ii) Changes of rainfall and temperature for the past 10 years and the impacts to the communities, and changes in farming system and crops as the adaptive capacity; and (iii) Food security, which included availability of sufficient food to last before the next harvest season, number of meals per day, types of food available in the household, and the measures taken when experiencing food shortage.

### Data analysis

The database was prepared using EpiData Version 3.1 software and data were entered using trained clerks. The data cleaning and quality check was done by a statistician who compared sample of the questionnaire with the entered data. Data was migrated to STATA (Stata Corps) for further analysis. Cross tabulations were done between selected variables of interest. Perception of individuals on climate change and their resilience strategies were assessed. The association between different factors was tested using *χ*^2^-test and proportional tests (where appropriate).

### Ethical considerations

The study obtained ethical approval from the Medical Research Coordinating Committee of the National Institute for Medical Research. Permission to conduct the study was sought from Kilosa District authorities. Oral informed consent was sought from the respondents after the objective and the methodology of the study had been explained. Privacy and confidentiality was maintained throughout the study. The respondents were assured of their right to withdraw from the interview at any time they would wish during the discussion.

## Results

### Socio-demographic characteristics of the respondents

A total of 399 individuals were interviewed. The age of respondents varied between 16 and 98 years with mean of 39.8 (SD = 15.5). Of these, females accounted for three quarters of the respondents. Most (62.41%) of the respondents had attained primary school education. Majority (91.23%) were involved in farming activities as the main occupation (Table 1).

**Table 1:** **Socio-demographic characteristic of respondents (N = 399)**

**Socio-demographic characteristics**		**Frequency**	**Percent (%)**
Sex	Male	103	25.81
	Female	296	74.19
Education Level	None	129	32.33
	Primary	249	62.41
	Secondary	21	5.26
Occupation	Farming	364	91.23
	Business	15	3.76
	Employee	5	1.25
	Other	15	3.76

### Knowledge of malaria

Awareness of the existence of malaria in the sample population was high. Majority (97.5%) of the respondents indicated to have heard of malaria. Three quarters (73%) reported that most people get sick from malaria during the rainy season. Nearly all (94.7%) knew that malaria is acquired through a mosquito bite, and virtually all (99.5%) confirmed that mosquitoes were present in their homes and in the surroundings. About 50% of the respondents felt that malaria had decreased in their village during the last 10 years, presumably leaving the other half unimpressed by malaria changes and mosquito abundance. The most commonly known symptom of malaria was fever (66.7%), followed by headache (44.4%), joint pains (43.6%), and nausea/vomiting (40.9%). Poorly known symptoms were diarrhoea (18.8%), convulsions (2.0%), and anaemia (1.5%).

Nearly all (95.5%) respondents mentioned the use of insecticide treated mosquito nets (ITN) as the main method of malaria prevention. Only a small proportion of the respondents mentioned other vector control methods including use of mosquito coils (4.8%); indoor residual spraying (6.8%); and larviciding (0.8%). Majority (78.7%) of the respondents believed that mosquito nets were the most effective method for preventing malaria. Environmental management of malaria was poorly known by the respondents. For instance, cleaning the environment around the homes was mentioned by 23.3% of the respondents while clearing grass and bushes around homes was mentioned by 12.5%. A large proportion (85.2%) of the respondents was of the opinion that reducing the population of mosquitoes would reduce malaria incidents.

Reported availability of ITN in the households was very high (98.90%), with an average of at least 2 nets per household. Almost all (96.0%) reported to have slept under a mosquito net the previous night. However, 17.0% of the respondents said there were some nights they did not sleep under a mosquito net. About 87.0% reported receiving at least one ITN for free from a government source, and 12% had received a subsidized net from the national voucher scheme. Slightly over a quarter (26.9%) reported to have bought at least one ITN, indicating that nets were a priority for these households. The majority (98%) of the respondents reported that they sought health care from government health facility when a household member fell sick. However, among them, 12.0% mentioned to seek care from drug stores. A few respondents (18.3%) admitted that sometimes they do not seek treatment from health facilities when someone in the household suffers from malaria. Of these, 4.8% reported not seeking treatment from health facilities because of the high cost.

### Livelihoods and malaria risk

Standing water (57.9%) and environmental factors including presence of grasses (42.9%) were the most frequently mentioned factors affecting mosquito abundance in and around homes. Climatic factors were not as commonly associated with malaria risk: only 35.8% mentioned amount of rainfall, 11.3% temperature, and 2.0% relative humidity. Although the respondents apparently knew that the state of the environment around the village contributes to mosquito breeding, they did not connect that standing water and grass growth are influenced by climatic events. Some 40.0% of the respondents did not know the linkages between crop farming and malaria prevalence. However, 30% of the respondents believed that rice farming contributed to malaria, and only 11% mentioned maize farming, which is the dominant occupation among the community members.

Over one-third of the respondents had outdoor activities starting before 5 am in the morning. Majority (92%) of the respondents admitted to wake up early in the morning to travel to their farms where they worked for the whole day. Travel time from homes to farms was approximated to take between one and three hours (~4-10 km). Over half (55.0%) of the respondents reported that there are times when they stay overnight in their farms, mostly during harvesting season. Staying overnight in the farms was described as necessary to reduce travel time to and from the farm, protecting crops from domestic and wild grazing animals as well as protecting crops from birds and insect pests. Encouragingly, 82.0% reported that they brought mosquito nets to protect themselves from mosquito bites while sleeping in the farms.

### Knowledge and perceptions on climate change

Only one in four understood the official Kiswahili term for climate change known as *Mabadiliko ya Tabia Nchi*. However, there was a general understanding that the rain patterns have changed in the past 10 years (less than 5% said there were no changes). There were a range of descriptions for this change: 35.0% said the rains begin earlier, while 21.0% said rains begin late; 22.0% said that there is more total rain than ever before, but 10% said there is less total rain; 38.0% described the rains as being more unpredictable.

**Figure 2:**
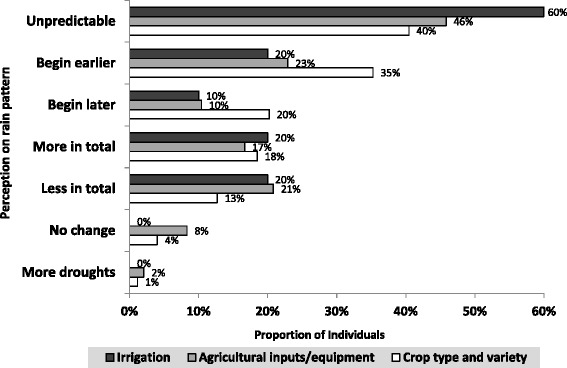
Community perception on rainfall pattern for the past 10 years and resilience in the farming practices.

Most of the respondents who described that the rain pattern was unpredictable adopted irrigation practices (60%) and about half (46%) initiated use of agricultural inputs and equipment, while 40% changed crop type and variety. The perception that rain began earlier was associated with changing crop type or variety. These differences were described more by male than female respondents (test of proportion, p-value < 0.001) (Figure 2).

**Figure 3:**
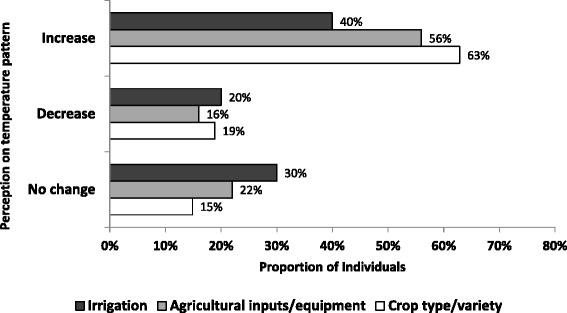
Community perception on temperature pattern for the past 10 years and resilience in the farming practices.

There was more agreement on temperature change, with 62.0% believing that the temperature has increased in the last 10 years (19.0% said there had been a decrease in temperature while 18.0% said there was no change). Most of the respondents who perceived that the temperature has increased changed crop type and/or variety (63%) while 56% started to use more agricultural inputs and equipment and 40% moved to irrigation practice. Similar to the changes in rainfall pattern, the impact of temperature changes was described more frequently by male than female respondents (test of proportion, p-value < 0.001) (Figure 3).

Respondents with primary education were more likely to perceive temperature increases than those with secondary or higher education, while those with secondary or higher education were more likely to be concerned with unpredictable rain patterns (Chi-square test, p-value <0.001). Older individuals (>45 years) were also more concerned with the impact of change in temperature and rain on the farm productivity than younger individuals (p-value <0.001). There was a tendency of families with large number of children to change their farming practice due to unpredictable rain or increase in temperature (p-value <0.01) (Table 2).

**Table 2:** **Proportion of respondents adopting changing in farming practices due to climate change by different socio-demographic characteristics**

	**Increase in Temperature**			**Unpredictable rain**		
	**Crop type/variety**	**Agricultural inputs/equipment**	**Irrigation**	**Crop type/variety**	**Agricultural inputs/equipment**	**Irrigation**
**Sex**						
**Male**	69%	65%	50%	47%	44%	50%
**Female**	61%	52%	38%	38%	47%	62%
**Education**						
**Primary**	71%	83%	100%	27%	40%	0%
**Secondary+**	59%	52%	33%	46%	47%	67%
**Age category (in years)**						
**<30**	55%	47%	40%	33%	33%	60%
**31 - 45**	63%	55%	50%	41%	56%	50%
**>45**	70%	69%	0%	49%	50%	100%
**Number of children**						
**<=3**	59%	48%	43%	41%	44%	57%
**>3**	67%	67%	33%	40%	48%	67%
**Overall**	**63%**	**56%**	**40%**	**40%**	**46%**	**60%**

Majority (86.0%) of the respondents reported to rely on weather patterns to determine planting time for their crops. Only about half of the respondents thought that the weather forecast by the Tanzania Meteorological Agency as provided through radio and television is useful in helping them to determine planting time.

It was observed that respondents who were relying on rain-fed crop production, employed different strategies to cope with extreme climate events such as drought. These included switching to more pest-resistant maize varieties, changing cultivars, seeking temporary jobs in urban areas or renting their fields. These were more reported as adaptation farming practices, indicating resilience to climate change. One in three had changed their crop variety in the last 10 years. About 16% had changed crop type and 11% had gained use of equipment (Figure 2). However, inputs and irrigation practices remained mostly unchanged (less than 5% reported the change). When asked about the impact of the 2009/2010 floods in Kilosa, 77% of the respondents reported their households to be affected. Some 30% reported to have been displaced from their homes, with others reporting being still displaced for the past two years. Farmland was heavily affected with 45% of the respondents reporting a permanent loss of part of their land.

### Food insecurity

**Figure 4:**
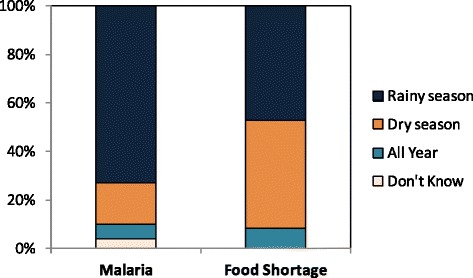
The proportion of respondents on the season when they experience most malaria and food shortage.

Food insecurity was described by the majority of respondents to be a common problem across the study area. Three quarters of the respondents reported that they had no sufficient production from their own farms to guarantee food security in their household for the year. Three quarters (73.0%) reported to having food shortages in their household in the past five years. About half said they most often experienced severe food shortage during the rainy season - the season when most households experience several bouts of febrile illnesses including malaria (Figure 4). This is also the planting season, when farmers need to put much of their energy in farming to ensure high productivity. Maize was as staple food for almost all (98.35%) respondents, meaning their household would be upset by poor maize harvests. About a quarter (26%) had less than two meals a day.

## Discussion

The community of Kilosa had at least some basic understanding of malaria, its causes, symptoms and prevention methods. They were apparently employing recommended prevention methods including use of mosquito nets and seeking treatment from health facilities. Similar findings have been reported in the district in another study [18]. Despite this knowledge and efforts to scale up malaria interventions, there was no consensus that malaria was declining among the farming communities. However, available statistics indicate that malaria has declined in all geographical zones of Tanzania. The prevalence of malaria parasitaemia among <5 years old children in Morogoro Region (where Kilosa District belongs) has fallen from 15.7% in 2008 to 7.4% in 2012 [19,20]. On the other hand, a recent community-based study in the district indicated a prevalence of 3.5% among schoolchildren; with those from Kimamba having the lowest prevalence [13].

This study hypothesized that some the effectiveness of malaria interventions, including mosquito nets, may be affected by rural farming lifestyles. One concern with effective mosquito net usage for populations such as Kilosa, where farms are located far from family homes, is resultant time spent out of the home during mosquito biting hours. This may occur when farmers need to leave their home before 5 am to reach their field early in the day, or when farmers spend the night in their field to cut down travel time or to protect their crops at harvest time. In these situations, even if members of the households have mosquito nets they will not be protected at all times. Indeed, in Kilosa district, the habit of rising before 5 am for daily farming activities was common, which makes mosquito nets ineffective for preventing mosquito bites for some time when mosquitoes are still biting. To their credit, the community proved resilient in taking extra measures to use their mosquito nets whenever possible during night farm activities. In fact, spending the night at the field may be a better practice than rising early to go to the farm and being exposed to mosquito bites during commute and when farming. The mosquito net campaigns, which have clearly been successfully adopted in Kilosa is evident from their widespread use [18,21]. The national malaria control programme should take this into consideration and make sure that ITNs are being used effectively in make-shift shelters.

Human activity outside the home into late evening and early morning hours is very common in Kilosa district. Outdoor human activities observed in this study have implication in malaria transmission and control. Studies have indicated that *Anopheles gambiae* complex readily seek hosts in outdoor venues [22]. The relevance of outdoor night biting behaviour of mosquitoes to vector suppression depends greatly on whether outdoor biting coincides with human outdoor activity. Previous studies have reported that host seeking activity of *An. gambiae* peaks around midnight [23]. Future studies of human behaviour in relation to livelihoods would provide important insights into human activity and other potential risk factors associated with outdoor biting. Although some respondents in this study claimed to bring mosquito nets when carrying out outdoor farm activities, it is obvious that the use of the nets is limited when one has to walk chasing animals grazing in his/her farmstead. Similar to our finding that most malaria incidence occur during the rainy season, in a neighbouring Kilombero Valley, the rainy season (which is also the main cultivation period, has been reported to be the time of high vulnerability for the farming population because of food insecurity, labour stress and poor access to health services [1]. In Côte d'Ivoire, disease episodes during the cultivation period have been described to result in lower crop yield and consequently lower household’s income [24]. The findings that farmers were carrying mosquito nets to their farms have also been reported [1] in the Kilombero Valley. Our findings and others in Tanzania, indicate that household members spend a considerable part of the farming season living in very basic shelters under difficult conditions and away from accessibility to health care services [1].

The high proportion of displaced residents and loss of farmland reported by the respondents pose problems to both malaria control efforts (including mosquito nets hung in the homes) and food security (from subsistence agriculture). Flood preparedness measures should address malaria control and sustainable food security. The lack of comprehension of climate change shows that the community has not received education on climate change, which could prepare them for possible consequences. There was a general awareness that the temperature was rising and the rains were changing [25], which may have attributed to some of the change in crop variety reported by the farmers. Such flexibility is important for resilience to climate change. Similar to our findings in Kilosa District, results from a study in north-eastern [26] and south-western highlands [25] of Tanzania also have revealed that most farmers were aware of ongoing climate variability.

In this study, food shortage among households in Kilosa was common. Similar findings have been reported in south-western Tanzania [25]. Managing seasonal climate risks for food production more effectively is challenging in Sub-Saharan Africa where highly variable rain fed environments and projected long-term increases aridity pose serious risks to agriculture [27]. Seasonal climate forecasts are an important planning tool at the farm level that can lead to better management of seasonal climate risks and instil processes useful to longer-term adaptation. However, in this study, majority of farmers were not aware of the usefulness of weather forecast. Reporting two or less meals increased the likelihood of food insecurity and malnutrition. Studies elsewhere have already shown that children who are malnourished are more susceptible to malaria morbidity and mortality [28,29]. In the current study, an important result is the seasonal nature of food insecurity and malaria problems in the district. The greatest time of need is during the rainy season, for both hunger alleviation and malaria prevention. Food insecurity and malnutrition seriously impedes efforts to control diseases in resource poor countries [30]. Previous studies have documented an increased risk of malaria associated with micronutrient, including zinc and vitamin A deficiencies [28]. Children living in severely food insecure households are more likely to experience micronutrient deficiencies, resulting in greater impairment in their malaria-protective immune function [28,29]. Besides its impact on physical health, household food insecurity and the poverty-related determinants of this condition have been associated with maternal stress and suboptimal psycho-emotional human development in diverse settings [10,31].

It has been described that food insecurity is common in developing countries. In Sub-Saharan Africa alone, an estimated two billion people experience food insecurity and its consequences like malnutrition and dietary deficiencies [32]. A high proportion of households in sub-Saharan Africa are therefore in a constant process to mitigate and cope with hunger or hunger threat in addition to communicable diseases. In Tanzania, it is estimated that 58% of the households are experiencing food insecurity problem [33] and thus their mitigation and coping strategies are seriously threatened by the high burden of communicable diseases. In rural Haiti, severe food insecure was a risk factor for perceived clinical malaria [10]. Malaria has been described to occur within the context of widespread poverty, poor health, and food insecurity.

Several limitations of this study restricted its comprehensiveness. Self-reporting could have resulted in recall bias. Convenience sampling may not have accurately represented the entire population, including men who often were not present in the home. There was missing input from farmers who were in the fields during the days surveying occurred.

## Conclusions

This study revealed information about malaria, climate change, livelihoods and food security, through the eyes of those whom policy makers seek to address. The results show a need for improvement in health, environment and agricultural sectors, with some overlapping issues that can be addressed in tandem. Policy implications stemming from this study involve a need for an integrated and intersectoral approach among experts in different fields that draws on community participation. Specifically, farmers need to be prepared in accordance with information about climate change and possible adaptations in order to ensure food security and economic stability in coming years. The findings of this study suggest that communities affected by malaria also commonly suffer from chronic food insecurity, and that both disorders affect small-scale farmers’ livelihoods. Given the tools and information about how to prevent malaria and improve their agricultural practices, farmers are willing to make changes to their livelihoods.

## Additional file

Additional file 1:
**Multilingual abstracts in the six official working languages of the United Nations.**
