# Supplementary material for: Knowledge, perception and practices about malaria, climate change, livelihoods and food security among rural communities of central Tanzania
Source: Infect Dis Poverty. 2015 Apr 24;4:21. doi: 10.1186/s40249-015-0052-2 (PMC4408568; doi:10.1186/s40249-015-0052-2)
Supplement: Additional file 1: — Multilingual abstracts in the six official working languages of the United Nations. [file 40249_2015_52_MOESM1_ESM.pdf]

Translation of the abstract into the six official working languages of the United Nations

انتشار عدوى الملاريا بين النساء الحوامل وعوامل خطورتها في مجتمع شمال غرب نيجيريا شبه المدني

ساني عبد الله فنا، محمد دنلادي أبو بكر بونزا، سول علي أنكا، آسيا عمر إمام وشيهو عثمان نتالا.

موجز

نبذة: تستمر عدوى الملاريا خلال فترة الحمل في تشكيل مشكلة صحية عمومية خطيرة ذات مخاطر كبيرة على الأم والجنين والوليد. تهدف هذه الدراسة إلى تحديد نسبة انتشار الملاريا وعوامل الخطر المحتملة لعدوى الملاريا بين الحوامل في المنطقة شبه المدنية في شمال غرب نيجيريا. فالحامل هي من بين الأفراد الأكثر قابلية للإصابة بعدوى الملاريا. وتشكل معرفة حالة إصابتها بعدوى الملاريا معياراً هاماً من أجل قياس فعالية أي برنامج لمراقبة الملاريا.

الطرائق: أجرينا دراسة لانتشار العدوى في منطقة أرغونغو شبه المدنية في ولاية كّبي في نيجيريا. شملت الدراسة مئتيّ وخمس وخمسين حاملاً بعد الحصول على موافقتهن الواعية المسبقة. وتم استخدام استبيان من أجل التحقيق في الموجز الاجتماعي الديمغرافي لبيانات كل حامل مشاركة ومرحلة حملها وموقفها من استخدام الناموسية المعالجة بمبيد حشري (ITNs). كما تمّ جمع عيّنات دم محيطي وأُعدت مسحات دم سميكة تمّ تلوينها بالغيمزا لفحصها لمعرفة احتمال إصابة الحامل بتسمم الملاريا الدموي الطفيلي. كذلك، تمّ تحليل الترابط بين العمر ومستوى التعليم واستخدام الناموسية المعالجة بمبيد حشري والإصابة بعدوى الملاريا خلال فترة الحمل من خلال استخدام اختبار خِي مربّع.

النتائج: كانت النتيجة أن 106 امرأة حامل (41.6%) من أصل 255 مصابات بطفيليات الملاريا بلغ متوسط كثافة الطفيليات لديها 800/μل. وتبيّن أن انتشار الطفيليات وكثافتها انخفضا مع التقدم بالعمر. وأشار اختبار خِي المربع أن الافتقار للتعليم وعدم استخدام الناموسية المعالجة بمبيد حشري كانا مرتبطين على نحو كبير بعدوى الملاريا.

الاستنتاج: لا تزال الملاريا تشكل مسألة صحية عمومية رئيسية بين الحوامل ويعود سببها الرئيسي إلى الأمية وعدم الالتزام باستخدام الناموسية المعالجة بمبيد حشري. ستساعد زيادة التوعية بشأن الإجراءات الوقائية المتعلقة بالملاريا والمشاركة المبكرة في خدمات رعاية الحوامل في تخفيض الإصابة بعدوى الملاريا وبالتالي، بتخفيض معدل الاعتلال والوفيات.

Translated from English version into Arabic by Liliane Hatem, through

TRANSLATORS
WITHOUT BORDERS

尼日利亚西北部半城市化社区孕妇疟疾感染的发病率和相关风险因素

撒尼 阿卜杜拉 发娜，穆罕默德 • 阿布巴卡尔， 但拉蒂 不扎，苏莱阿利尤安卡，阿希亚 欧麦尔伊玛目，谢胡乌斯曼 纳塔拉

摘要

引言：孕期疟疾感染是一个严重的公共卫生问题，威胁着母亲及其胎儿和新生儿的健康。本研究的研究目的是确定尼日利亚西北部半城市化社区中孕妇疟疾的流行程度和潜在的感染风险因素。孕妇是疟疾感染的高发人群。对疟疾感染情况的认知程度是衡量疟疾防控项目有效性的重要指标。

方法：在尼日利亚凯比州阿尔贡古的一个半城市化地区进行了一个横断面研究，共调查 255 位孕妇，均已知情同意。应用调查问卷调查社会人口概况、妊娠阶段和对使用经杀虫剂处理的蚊帐（INTs）的态度。采集外周血标本，制作厚血膜涂片，吉氏染色后进行镜检疟原虫。采用卡方检验分析妊娠期间疟疾感染与年龄、教育程度和 INTs 使用情况的相关性。

结果： 接受调查的 255 位孕妇中，106 位感染了疟原虫，平均密度为 800 个/μl。疟疾患病率和寄生虫密度均随着年龄的增长而下降。卡方检验表明，缺乏教育和未使用驱虫蚊帐与疟疾感染显著相关。

结论：由于缺乏教育、不合规使用蚊帐，疟疾仍是孕妇人群中一个重要的公共卫生问题。因此，提高人们对疟疾预防措施的认识和提早进行产前保健将有助于减少疟疾，也相应地降低其发病率和死亡率。

Translated from English version into Chinese by Chen Jin, edited by Yang Pin, through

CDC CHINESE CENTER FOR DISEASE CONTROL AND PREVENTION
NATIONAL INSTITUTE OF PARASITIC DISEASES

Prévalence et facteurs de risque associés à l’infection palustre des femmes enceintes dans une communauté semi-urbaine du nord-ouest de Nigeria

Sani Abdullahi Fana, Mohammed Danladi Abubakar Bunza, Sule Aliyu Anka, Asiya Umar Imam, Shehu Usman Nataala

RÉSUMÉ

Contexte : Le paludisme pendant la grossesse reste un grave problème de santé publique, qui entraîne des risques considérables pour la mère, le fœtus et le nouveau-né. Le but de cette étude était de déterminer la prévalence du paludisme et les facteurs de risque possibles d’infection palustre chez les femmes enceintes dans une communauté semi-urbaine du nord-ouest de Nigeria. Les femmes enceintes font partie des personnes les plus sensibles à l’infection par le paludisme. La connaissance de leur stade d’infection palustre est un critère important pour mesurer l’efficacité d’un programme de lutte antipaludique.

Méthodes : Nous avons mené une étude transversale dans la zone semi-urbaine d’Argungu, dans l’État de Kebbi au Nigéria. Deux cent cinquante-cinq femmes enceintes ont été incluses dans l’étude après que leur consentement oral a été obtenu. Le profil sociodémographique de chaque participante, son stade de grossesse et sa disposition à utiliser des moustiquaires traitées avec un insecticide ont été examinés au moyen d’un questionnaire. Des échantillons de sang périphérique ont été prélevés et des frottis de sang en goutte épaisse ont été préparés et colorés avec des colorants de Giemsa pour vérifier la parasitémie palustre. Les liens entre l’âge, le niveau d’éducation et l’utilisation de moustiquaires traitées, d’une part, et d’autre part l’apparition d’une infection palustre pendant la grossesse ont été analysés en utilisant le test du chi-deux.

Résultats : Cent six femmes (41,6 %) parmi les 255 femmes enceintes étaient infectées par le paludisme, avec une densité parasitaire moyenne de 800 parasites/µl. Nous avons découvert que la prévalence et la densité parasitaire diminuaient quand l’âge augmentait. Le test du chi-deux a indiqué que le manque d’éducation et la non utilisation de moustiquaires traitées avec un insecticide étaient liés de manière significative à l’infection palustre.

Conclusion : Le paludisme reste un problème majeur de santé publique pour les femmes enceintes, principalement à cause de l’illettrisme et la non observance de l’utilisation de moustiquaires traitées avec un insecticide. Sensibiliser aux mesures de prévention du paludisme et à la fréquentation précoce des service de soins anténataux aidera à réduire le paludisme et, par conséquent, la morbidité et la mortalité qui lui sont associées.

Translated from English version into French by Suzanne Assenat, through

TRANSLATORS
WITHOUT BORDERS

Распространенность и факторы риска, связанные с малярийной инфекцией у беременных женщин в полугородском сообществе северо-востока Нигерии

Сани Абдуллани Фана, Мохаммед Данлади Абубакар Бунза, Суле Алию Анка, Асия Умар Имам, Шену Усман Натаала

АННОТАЦИЯ

Предпосылки: Малярия во время беременности остается серьезной проблемой общественного здравоохранения при существенных рисках для матери, плода и новорожденного. Целью настоящего исследования было определение распространенности малярии и возможных факторов риска малярийной инфекции среди беременных женщин в полугородском районе на северо-востоке Нигерии. Беременные женщины являются одной из групп, наиболее подверженных малярийной инфекции. Знание их статуса малярийной инфекции является важным подспорьем для измерения эффективности любой программы контроля малярии.

Методы: Нами было проведено перекрестное исследование в полугородском районе Аргунгу (штат Кебби) в Нигерии. В исследование после получения устного информированного согласия было включено 250 беременных женщин. С помощью вопросника для каждого участника выяснялись социо-демографический портрет, стадия беременности и отношение к использованию сеток, обработанных инсектицидом. Были собраны образцы периферической крови и подготовлены толстые мазки крови, окрашиваемые красителем Гимза для выяснения паразитемии малярии. С помощью критерия хи-квадрат анализировалась зависимость между возрастом, уровнем образования и использованием сеток, обработанных инсектицидом, и частотой распространения малярийной инфекции во время беременности.

Результаты: 106 из 255 беременных женщин (41,6%) были инфицированы малярийными паразитами при средней плотности паразитов 800 паразитов/мкл. Было обнаружено, что распространенность и плотность паразитов уменьшаются с повышением возраста. Анализ на основе критерия хи-квадрат показал, что недостаток образования и неиспользование сеток, обработанных инсектицидом, в значительной степени связаны с инфицированием малярией.

Вывод: Малярия среди беременных женщин все еще является большой проблемой общественного здравоохранения в основном из-за неграмотности и несогласия использовать сетки, обработанные инсектицидом. Повышение знакомства с мерами предотвращения малярии и раннее участие службы дородового наблюдения помогут уменьшить число случаев малярии и, как следствие, связанные с ней коэффициенты заболеваемости и смертности.

Translated from English version into Russian by Alexander Somin, through

TRANSLATORS
WITHOUT BORDERS

Prevalencia y factores de riesgo asociados al paludismo entre las mujeres embarazadas de una comunidad semiurbana de Nigeria noroccidental

Sani Abdullahi Fana, Mohammed Danladi Abubakar Bunza, Sule Aliyu Anka, Asiya Umar Imam, Shehu Usman Nataala

RESUMEN

Antecedentes: El paludismo en el embarazo sigue siendo un grave problema de salud pública, que acarrea un riesgo considerable para la madre, el feto y el recién nacido. El objeto del presente estudio ha sido establecer la prevalencia del paludismo y los posibles factores de riesgo de la infección entre las mujeres embarazadas de una zona semiurbana de Nigeria noroccidental. Las mujeres embarazadas se encuentran entre la población más predispuesta a la infección por paludismo. Un criterio importante a la hora de cuantificar la eficacia de cualquier programa de control del paludismo es conocer la situación de la infección.

Métodos: Hemos realizado un estudio transversal en la zona semiurbana de Argungu, en el estado de Kebbi en Nigeria. Se han incluido en el estudio doscientas cincuenta y cinco mujeres embarazadas que dieron su consentimiento verbal informado. Se ha investigado mediante cuestionario el perfil sociodemográfico, la fase de embarazo y la actitud hacia el uso de mosquiteras tratadas con insecticidas (ITN) de cada participante. Con el fin de comprobar la densidad parasitaria, se han recogieron muestras de sangre periférica y prepararon gotas gruesas para la tinción de Giemsa. La relación entre edad, nivel educativo y uso de ITN e incidencia del paludismo durante el embarazo se ha analizado utilizando la prueba de la χ2.

Resultados: Ciento seis (el 42%) de 255 mujeres embarazadas estaban infectadas con parásitos del paludismo, con una densidad parasitaria media de 800 parásitos/µl. Se ha descubierto que la prevalencia y la densidad parasitaria disminuyen conforme aumenta la edad. La prueba de la χ2 evidenció que la falta de educación y la no utilización de ITN guardaban una estrecha relación con la infección por paludismo.

Conclusión: El paludismo todavía es un gran problema de salud pública entre las mujeres embarazadas, principalmente a causa del analfabetismo y de la falta de uso de ITN. El aumento de la sensibilización sobre las medidas preventivas contra el paludismo y una atención precoz en el servicio de asistencia prenatal ayudarán a reducir la infección y, por consiguiente, su morbimortalidad asociada.

Translated from English version into Spanish by Raquel Bentué, through

TRANSLATORS
WITHOUT BORDERS
